# Characteristics, resource consumption and outcome of cancer patients admitted to ICUs

**DOI:** 10.1186/cc11019

**Published:** 2012-03-20

**Authors:** R Garcia, L Terceros, I Saez, J Flordelis, L Colino, C Mudarra, S Temprano, J Montejo

**Affiliations:** 1 12 de Octubre Hospital, Madrid, Spain

## Introduction

The development of cancer treatment has improved the prognosis for cancer patients and they need more support measures in the ICU. Our objective is to evaluate the characteristics and evolution of cancer patients admitted to a general ICU of a university hospital.

## Methods

A retrospective study of cancer patients admitted to an ICU from January 2008 to December 2010. We collected demographic and cancer characteristics, reason for admission, complications, resource consumption and mortality. We compared quantitative variables with the Student *t *test and the qualitative variables with the chi-square test, statistical significance accepted as *P *< 0.05.

## Results

A total of 108 patients were admitted with cancer, 23 with cured cancer were excluded, so we selected 85 patients (4.38% of total admissions). Sixty-eight percent were male, with a mean age of 60.21 ± 14.31 years and with an APACHE score of 22.21 ± 9.13. Solid cancer was more frequent, 76.6% (urogenital 20%, lung 15.4% and low intestinal 15.4% were the most common). In the hematologic cancers (23.5%), the most frequent were non-Hodgkin lymphoma and acute leukemia (both 7%). Active cancer (new diagnosis, recurrence or progression) was presented in 75.3%. The main reason for admission was respiratory failure (52.9%), shock (18.8%) or neurological impairment (16.5%). The most common diagnoses were pulmonary sepsis (23.5%), other sepsis (21.2%) and heart failure (8.2%). The ICU stay was 7.20 ± 12.32 days; with a mortality of 41.2% (hospital mortality 50.6%). The mortality was higher in the active disease (91% vs. 64%), *P *< 0.01. Patients who died developed more respiratory (88.6% vs. 48%), hemodynamic (91.4% vs. 44%), renal (68.6% vs. 16%) or hematologic failure (45.7% vs. 16%), *P *< 0.03. Septic patients were those with higher ICU mortality (55.3% vs. 29.8%) and hospital mortality (63.2% vs. 40.4%), *P *< 0.05. By contrast, the patients with the longest survival were the neurological (90% vs. 54.7%) and cardiology patients (88.9% vs. 55.3%), *P *< 0.05. Patients who died needed more MV (88.6% vs. 52%), vasopressors (91.4% vs. 46%) or dialysis (34.3% vs. 4%), *P *< 0.01. The hematologic cancer had more cardiovascular (85% vs. 56.9%) or hematologic failure (65% vs. 16.9%) and neutropenia (45% vs. 9.2%) with *P *< 0.03, but this is not reflected in more consumption of resources or mortality.

## Conclusion

The mortality was associated with organ failure and greater need for resources. Hematologic cancer develops more organ failure without affecting resource consumption or their outcome in our series. Septic patients have higher ICU and hospital mortality, and neurological patients lower.

